# A pathway of nanocrystallite fabrication by photo-assisted growth in pure water

**DOI:** 10.1038/srep11429

**Published:** 2015-06-16

**Authors:** Melbert Jeem, Muhammad Rafiq Mirza bin Julaihi, Junya Ishioka, Shigeo Yatsu, Kazumasa Okamoto, Tamaki Shibayama, Tomio Iwasaki, Takahiko Kato, Seiichi Watanabe

**Affiliations:** 1 Graduate School of Engineering, Hokkaido University, N13, W8, Kita-ku, Sapporo, Hokkaido 060-8628, Japan; 2 Faculty of Engineering, Hokkaido University, N13, W8, Kita-ku, Sapporo, Hokkaido 060-8628, Japan; 3 Hitachi Research Laboratory, Hitachi Ltd., 7-1-1 Omika, Hitachi, Ibaraki, 319-1292, Japan

## Abstract

We report a new production pathway for a variety of metal oxide nanocrystallites via submerged illumination in water: submerged photosynthesis of crystallites (SPSC). Similar to the growth of green plants by photosynthesis, nanocrystallites shaped as nanoflowers and nanorods are hereby shown to grow at the protruded surfaces via illumination in pure, neutral water. The process is photocatalytic, accompanied with hydroxyl radical generation via water splitting; hydrogen gas is generated in some cases, which indicates potential for application in green technologies. Together with the aid of *ab initio* calculation, it turns out that the nanobumped surface, as well as aqueous ambience and illumination are essential for the SPSC method. Therefore, SPSC is a surfactant-free, low-temperature technique for metal oxide nanocrystallites fabrication.

New approaches to manufacturing the nanocrystallites of metallic oxides are desired due to their emerging applications in a wide range of high-technology applications^1^,^2^,^3^,^4^,^5^,^6^,^7^,^8^. In the case of liquid-solid phase crystallisation studies, the surface morphology control plays an essential role in influencing the nucleation of nanocrystallites^9^. Recent studies have demonstrated the role of surface chemistry and morphology utilizing various mechanisms gained from polymeric substrates^10^,^11^. Achieving those understandings provides a powerful means to widespread reports in nanocrystallites research.

Our interest is in the easy feasible design of nanocrystallites fabrication beneficial for the nanotechnology and environment. With regard to obtaining the desired products, we herein report a new pathway of production for variety of metal oxides nanocrystallites via SPSC. We chose various metals (Zn, W, Cu and Ce) to demonstrate the effectiveness of the submerged photosynthesis of crystallites (SPSC) method in obtaining a variety of morphologies of metal oxide nanocrystallites (NCs). The present report primarily focuses on ZnO nanofabrication due to its promising environmental^12^ applications and broad range of modern device applications, including light-emitting diodes^13^, photo-detectors^14^, gas sensors^15^, and solar cells^16^.

The functions of these semiconductive ZnO (with a wide bandgap of 3.37 eV) devices are crucially dependent on the nanostructure morphology. Hence, it is important to tune and stabilise the syntheses parameter for improved performance. To this end, there has been a substantial increase in the number of reports on hydrothermally synthesised ZnO nanostructures^17^,^18^, including lasing^1^ and the addition of metal-ion impurity^19^ techniques. Expanding on these efforts, this study develops a rational and environmentally benign approach to synthesise a plethora of ZnO nanomorphologies.

We employed our initial metal surface treatment based on the utilisation of the submerged liquid plasma process^20^,^21^. The reaction of plasma in an aqueous solution facilitates the synthesis of metal oxide powder^22^,^23^. Hence, we adopted this technique for the direct (one-step) synthesis of ZnO “seeds”. Our intention was to create a semiconductive reformed layer with protruding characteristics (nanobumps) on the material. For this purpose, a raw Zn metallic plate was used as the target material for the formation of nanobumps. The NC growth was then completed by a “photosynthesis” reaction, where the irradiation of UV light (typically λ = 365 nm and I = 28 mWcm^−2^) on the nanobumps inside pure water assisted the growth of ZnO NCs.

The SPSC method is different from previous nanofabrication reports^24^,^25^, in which a hydrothermal decomposition process using UV-Vis light irradiation directed the formation of NCs. Instead, we found that the illumination in ultrapure water induced an apical growth characteristic via water radiolysis, as will be described later. Furthermore, we were able to demonstrate working NC fabrication at room temperature (RT) under surfactant- and contamination-free conditions by eliminating the need for organometallic or other organic solution phases. Similar to the growth of green plants via botanical photosynthesis, our SPSC method requires only light, water and nanobumps (crystallite seeds). Interestingly, the SPSC end products were accompanied by the generation of hydrogen gas, which gives rise to a potential application of this methodology as a green technology for energy, chemistry and nanotechnology.

## Results

**Figure 1:**
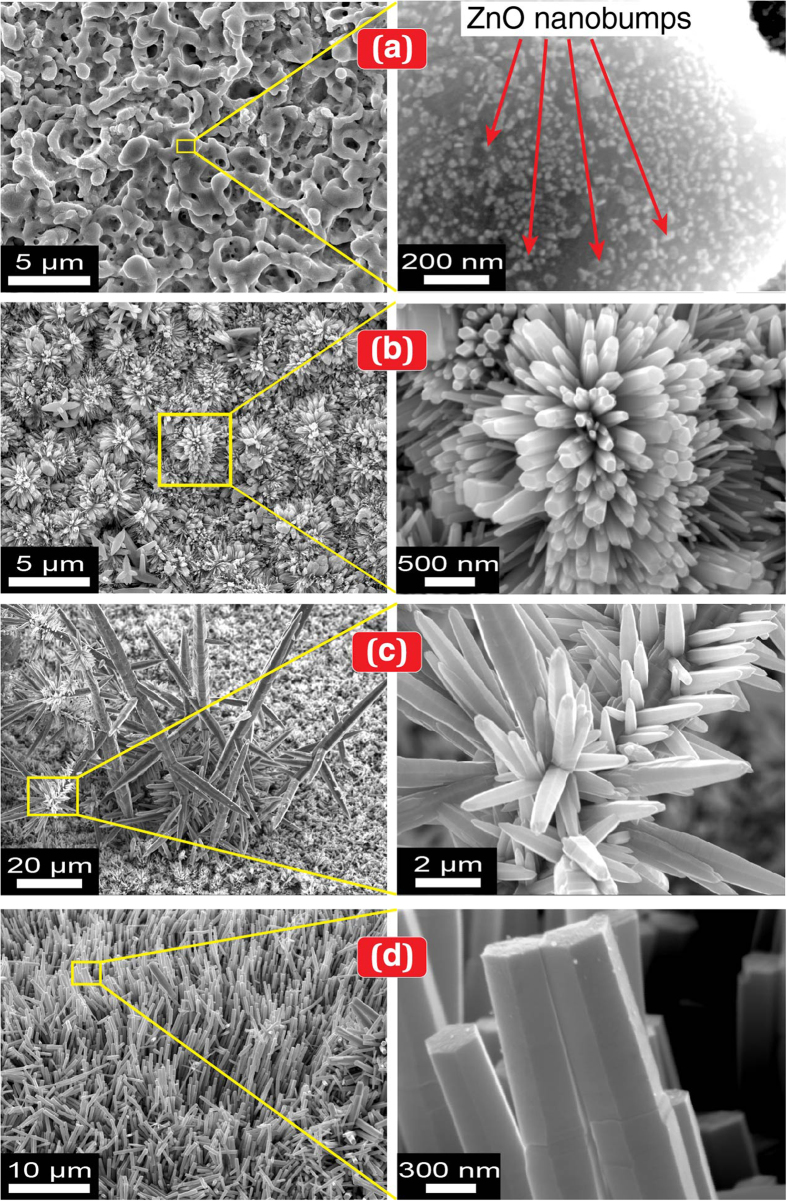
Surface morphology after SPSC on a Zn substrate plate. (**a**) ZnO nanobumps (plasma 140 V, 10 min, UV_0h_). (**b**) ZnO nanoflowers (UV_24h_). (**c**) ZnO dendrites (UV_24h_). (**d**) ZnO nanorods (UV_72h_). The right panel images are the respective magnified FE-SEM micrographs. The heterogeneous growth is due to the local morphology variation via the plasma treatment. Typically, a fine structure of NCs can be obtained at room temperature after 24 h of UV irradiation. Extended irradiation increased the size and diameter but terminated the apical growth to yield flat, hexagonal tip ends (at UV_72h_ irradiation, as shown in (**d**) and Supplementary Fig. S2).

**Figure 2:**
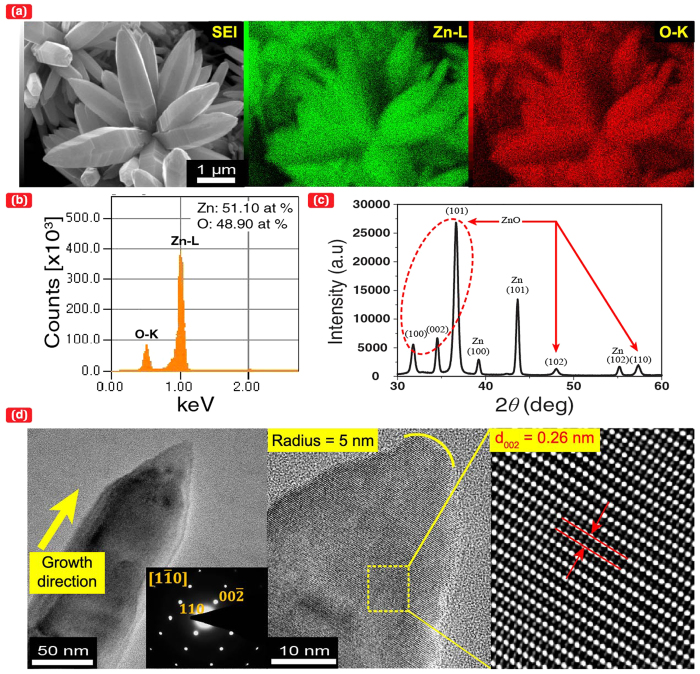
Structural characterisation of ZnO NCs. (**a**) 2-D EDS map of ZnO nanoflowers (UV_48h_); the left panel is a secondary electron image of ZnO crystals grown on the Zn substrate, centre: Zn, and right: O. (**b**) EDS spectrum and quantitative composition of ZnO. (**c**) XRD pattern of Zn and ZnO NCs. (**d**) TEM micrograph of a ZnO nanorod on a carbon thin film; the left panel is the TEM image (200 × 200 nm) of the ZnO nanorod, and the inset is the SAED pattern obtained along the 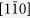 direction; the centre and right panels are the HRTEM image (40 nm × 40 nm) and its magnified image after inversed Fourier transformation, respectively. Fig. 2(d) exhibits the apical growth direction of ZnO in the c-axis.

Figure 1(a) presents a scanning electron microscopy (SEM) image of ZnO nanobumps that were tailored by submerged liquid plasma treatment and depicts the metal surface having average of two or more protrusions per 10 μm^2^. The protruded surface exhibits an average diameter of 1 μm or less. The higher-magnified image indicates the small ZnO seeds homogeneously localised on the protruded surface; these seeds have an average diameter of approximately 20 nm. Here, a drastic change in the seed clustering was observed after subsequent UV irradiation in ultrapure water and ambient temperature: widely spread ZnO NCs covered the metal substrate. The previously localised seeds grew outward to form a bunch of nanorods that formed into nanoflowers (dandelion-like), dendrites (tree-like), and aligned nanorods (lawn grass-like) (Fig. 1(b–d), respectively). The SEM-energy-dispersive X-ray spectroscopy (EDS) analysis (Fig. 2(a,b)) and X-ray diffraction (XRD) analysis (Fig. 2(c)) for the ZnO NCs illustrated that the NCs were synthesised on a Zn substrate, which contained Zn and O. As evidenced by the selected area electron diffraction (SAED) pattern and high-resolution transmission electron micrograph (HRTEM) of a nanorod examined along the 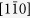 axis (Fig. 2(d)), the nanorods were single crystallites (a wurtzite structure). The apical growth direction was in the c-axis <001>. This result is consistent with previous ZnO crystal growth reports^24^,^26^,^27^.

## Discussion

**Figure 3:**
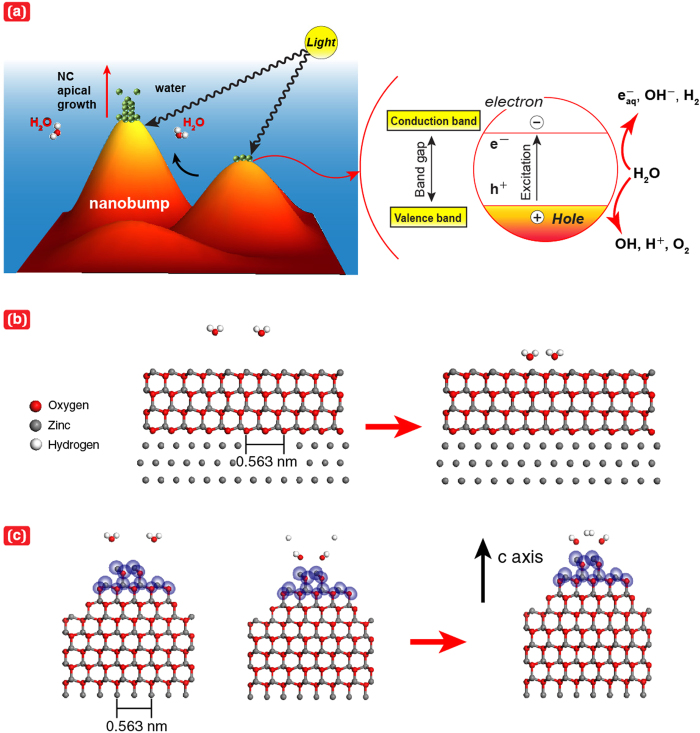
Nanobumped surface effect in the SPSC model and simulation by molecular dynamical *ab initio* calculations. (**a**) A schematic of SPSC with a nanobumped surface. (**b**) H_2_O molecules with a flat surface of ZnO. Water molecules localise at stable places near the flat surface. (**c**) Water molecule stabilised on the top of a nanobump (radius of curvature, R = 0.5 nm). Electron density isosurfaces of 2.0 electron/Å^3^ (purple coloured) are observed only near the top of a nanobump. The state was relaxed to a state where a H_2_ molecule is formed by equilibrating the state with the dissociation of H_2_O molecules into OH and H.

To consider the SPSC mechanism (as illustrated in Fig. 3(a)) via dissociation of water molecules (H_2_O) on nanobumped ZnO surfaces, the electron density and bond-dissociation energy required to alter H_2_O into OH and H radicals were calculated using *ab initio* simulations^28^,^29^. To clarify the effect of the apical growth of NCs accompanied by the dissociation of water, the bond-dissociation energy was calculated for the flat surface (Fig. 3(b)) and nanobumped surface (Fig. 3(c) for curvature radius, R = 0.5 nm). The high electron density appeared to be localised near the top surface of a nanobump (Fig. 3(c)). The dissociation energy for each surface was evaluated as the difference in the total energy of the dissociated state with H and OH and that of the equilibrated bonded state of H_2_O. The calculated value for the flat surface was 5.03 eV without considering the photoexcitation effect, corresponding to the experimentally measured direct dissociation energy of a water molecule (5.1 eV)^30^. In contrast, the dissociation energy for the nanobumped surface model was 0.323 eV when R = 0.5 nm (subsequently, 0.409 eV for R = 1.0 nm and 0.552 eV for R = 2.0 nm). Notably, the dissociation energy of a water molecule was lower for the nanobumped surface. Overall, the local electron density and dissociation energy reduction at the top of the NC bumps played a key role in the SPSC process.

We now suggest that the mechanism for photosynthesised NCs in water via the SPSC process (Fig. 3(a)) can, *in principle,* be described by the following photo-induced reactions:

The mechanism starts with water splitting into ion species in the vicinity of a semiconductive (SC) surface:


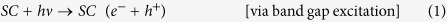



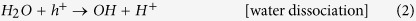



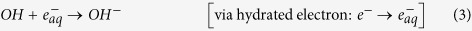


Hence, the water splits into ions by photoinduction (1)–(3):


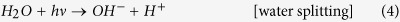


The formation of transient species (H, OH, 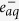) and other molecular byproducts (H_2_, H_2_O_2_) (see Fig. 3(c) for *H* + *H* → *H*_2_) can be well understood as water radiolysis in radiation chemistry^31^. These reactions occur in short times of less than micro-second-order. Presumably, assisted by the aforementioned morphology effect, *H*^+^ and *OH*^−^ ions are then locally separated, e.g., *H*^+^ at a valley in equation (2) and *OH*^−^ at an apical hill in equation (3) throughout a protruded surface. Otherwise, H_2_O will be immediately reproduced in the reverse of equation (4). Such locality-assigned ion production gives rise to a local pH imbalance: alkaline at the hill and acidic near the valley. Therefore, one can expect NC growth at a hill in association with hydrothermal reactions for ZnO generation in an alkaline solution^26^,^27^:


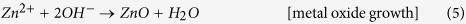


The metal may resolve into an ion at the valley:


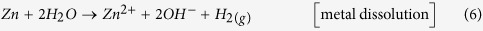


Hence, the net SPSC reaction is completed with


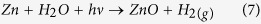


Similarly to known hydrothermal mechanism, NC growth by equations (5)-(6), might be accompanied by aggregation and recrystallisation processes of metal oxide nanoparticles. Thus, the SPSC is completed with three *principles*. Firstly, a photo-induced water splitting process. Secondly, separation of *H*^*+*^ and *OH*^*−*^ ions due to nanobumps protruded surface. Finally, aggregation and recrystallisation of metal oxide nanoparticles (superimposed hydrothermal reactions) result in the nanocrystallites growth.

**Figure 4:**
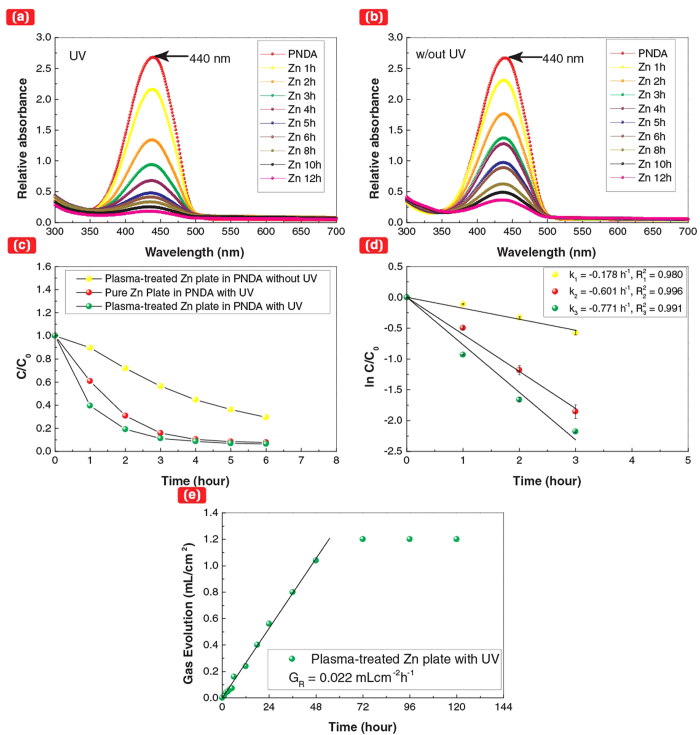
Detection of OH radicals and measurement of gas evolution. (**a**-**b**) PNDA peak intensity decrease at λ = 440 nm to observe the effect of UV irradiation. (**a**) Plasma-treated Zn plate (UV). (**b**) Plasma-treated Zn plate (No UV). (**c**,**d**) Decomposition trend for PNDA by evaluating the concentration change at the λ = 440 nm peak. (**e**) Time dependence of the gas evolution on extended UV-irradiated, plasma-treated Zn. The gas volume increase slowed down after UV_72h_, which is consistent with the terminated apical growth of a nanorod in Fig. 1(d).

The SPSC process characteristically predicts hydrogen gas (*H*_2(*g*)_) and hydroxyl radicals (OH) as intermediate products (see Fig. 4). To confirm our model, we detected H_2_ gas using gas chromatography (GC) after the SPSC experiment on Zn. The ratio of H_2_/O_2_ in the collected gas was evaluated to be nearly 10. H_2_ gas can also be produced by a typical photocatalytic reaction^12^ (2*H* + 2*e*^−^ → *H*_2_), as well as via water radiolysis (with hydrogen radicals, Fig. 3(c)) and hydrothermal reaction (equation (6)). In the present study, OH radical generation was also investigated (Fig. 4(a–d)) by monitoring the bleaching of *p*-nitrosodimethylaniline (PNDA) as the intensity of the characteristic absorption peak at λ = 440 nm decreased^32^,^33^. These results confirm the photochemical reactions proposed for the SPSC process, in which H_2_ gas and OH radicals were generated during the photo-assisted growth of ZnO nanorods.

In general, hydrothermal reactions in equations (5) and (6) are known to occur in alkaline solutions at higher temperatures^18^,^26^,^27^. Noting this, we tested the SPSC process at two different water temperatures (10 °C and 60 °C) other than room temperature. The NC growth was clearly enhanced at higher water temperatures. Nonetheless, the overall SPSC process is to occur assumed in a pure, neutral water environment rather than in acidic or alkaline aqueous solutions. However, one can observe the pH dependence or impurity additive influence on the final NC morphology. Moreover, the photocorrosion effect^34^, e.g., 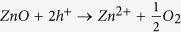, which causes ZnO to redissolve into Zn ions or *Zn*(*OH*)_2_ or 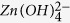 (equation (7)) and 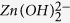 (equation (6)). They may suppress the reaction processes and thus reduce the yield of SPSC in equation (7). However, the re-dissolution effect results in the possibility of ZnO recrystallisation at the initial fine nanocrystallites to regrow into thicker and wider NCs by re-precipitation, as shown in Fig. 1(b–d).

**Figure 5:**
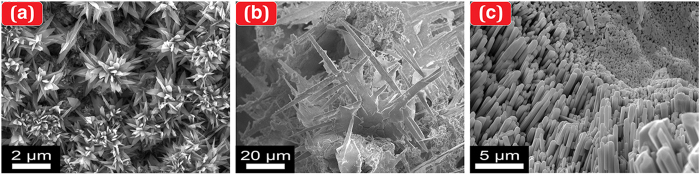
Various nanostructures of metallic oxides produced by the SPSC method. (**a**) CuO nanoflowers (UV_24h_). (**b**) CeO_2_ dendrites (Vis_24h_, λ ≈ 500 nm). (**c**) WO_3_ nanorods (UV_48h_).

With a slight modification of reactions in equations (5, 6, 7), our SPSC method can also be applied to derive a wide variety of metal oxide NCs (e.g., metals of Cu, Ce, and W, as shown in Fig. 5). These modifications revealed the difference in the SPSC morphology, resulting in various NCs. In the present work, we typically employed the submerged liquid plasma technique as an initial surface nanostructure treatment of natively oxidised NC seeds. Nevertheless, other possible alternative seeding methods can also be applied, including laser processing, ion irradiation, and tribological, mechanical scratching.

In summary, SPSC requires *light*, ranging from UV to visible (as shown in Fig. 5(b)), to assist the apical growth of NCs. Secondly, the use of *water* (specifically, ultrapure water) will deliver a fine structure of NCs. Because the NC morphology is sensitive to pH changes and water impurities, the additive effect resulted in different morphologies. For example, Si from tap water resulted in sphere-like crystallites and NaCl from natural seawater resulted in plate-like crystallites. These results are presumably caused by the alteration of the electronic state of the apical surfaces. Finally, metal oxide surface nanobumps act as the NC *seeds* and enclose the nucleation for the apical growth reaction.

## Methods

### Surface pretreatment

In the submerged liquid plasma experiment devices (Supplementary Fig. S1(a)), the anode was a φ0.5 × 1 mm platinum wire (Nilaco, Tokyo, Japan) with purity of 99.9% arched into a hemispherical glass mesh (R = 30 mm). The cathode (target material) was a raw metal Zn plate (Nilaco, Japan, 99.5%), cut into a size of 35 × 5 × 1 mm. A 60 mm^2^ contact area with a wrapped φ0.5 mm Cu wire (Nilaco, Japan, 99.9%) on the tip of the Zn plate was used to prepare the working electrode. A solution of 0.1 mol/l K_2_CO_3_ with pH 11.5 was used as the electrolyte. Deionised water was used as the washing solution. Prior to the experiments, both of the electrodes were washed with deionised water, and the electrolyte was preheated to 90 °C. Insulation of the contact area between the Cu wire and Zn plate was achieved by a φ10 mm glass tube, ensuring that the exposed Zn plate length was approximately 25 mm. Then, both of the electrodes were immersed in the K_2_CO_3_ solution (300 ml) and separated by distance of 30 mm. A discharge voltage of 140 V (current: 1.6–1.8 A) was applied across the electrodes using a direct current power supply (KIKUSUI, PWR1600H, Japan). The synthesis of nanobumps using submerged liquid plasma was conducted for a fixed reaction time of 10 min, appreciating the simple and time- and cost-efficient technique. At the end of the plasma reaction, the cathode was collected and washed with deionised water, and the length was cut to 25 mm. A white film surface, confirmed to constitute of ZnO, was obtained on the electrode surface. For further experimentation and analysis, the specimen was allowed to dry at ambient temperature.

### SPSC experiment

In the UV irradiation experiment (Supplementary Fig. S1(b)), the plasma-treated Zn plate was inserted into a polymethylmethacrylate (PMMA) cuvette, which was then filled with 4 ml of ultrapure water (Wako Pure Chemical, pH 7–7.5, resistivity 18 MΩ) and capped. Prior experiments, the ultrapure water was degassed to remove the dissolved gas. A UV lamp (UVP, B-100AP, USA) with 100 W longwave UV (λ = 365 nm, 3.4 eV) was mainly used for SPSC. Visible light irradiation (λ ≈ 500 nm) was employed for Fig. 5(b) using spot light source (Hamamatsu LightningCure LC8, L9588, Japan). The irradiance orientation was set to the horizontal position, and the distance between the specimen and UV lamp was set to 100 mm. In the typical synthesis of nanocrystallites, the UV irradiation was performed in a dark chamber for a fixed reaction time (24 h) at room temperature. Extended UV irradiation times (48 h and 72 h) were also applied to clarify the NC growth characteristics (Fig. 1(d), Fig. 5(c), and Supplementary Fig. S2). At the end of the UV irradiation, the specimen was collected, and the ultrapure water pH change was recorded using a pH meter (Horiba, D-51). For ZnO, the final pH of the water solution exhibited a typical increase to 8.5 in ambient temperature. The final water temperature increase was measured to be less than 10 °C.

### Crystallite characterisation

The surface morphology and elemental composition analysis of the substrates were monitored using a field emission scanning electron microscopy (FE-SEM, JEOL, JSM-7001FA). The chemical properties analysis was performed using X-ray diffraction (XRD, Rigaku, Tokyo, Japan, RINT2500HLB) with a Cu Kα line of 1.5406 Å and a scanning field of 2.5° ≦ 2θ ≦ 100°. Peak fitting was performed in referenced to JCPDS card 4-0831 and 5-0664. TEM micrographs, SAED patterns and HRTEM micrographs for the NCs were obtained using a double Cs-corrected-TEM (FEI, Titan cubed) operated at 300 kV.

### *Ab initio* calculation

The simulation models of a flat surface and a nanobumped surface were constructed using Materials Studio® atomic simulation software (Accelrys Software Inc.). The calculations were performed based on density functional theory (DFT)^35^,^36^. The radii of curvature of the nanobumped surface, shown in Fig. 3(c) and Supplementary Fig. S3(a) and S3(b), were set to 0.5, 1.0, and 2.0 nm, respectively. The grey, red, and white spheres in these figures represent zinc, oxygen, and hydrogen atoms, respectively. Initially, two free H_2_O molecules were placed in positions where strong interatomic forces were not exerted on each atom. The position of each atom in the equilibrated state was obtained using a dynamic simulated annealing method^28^,^29^. In this method, the electronic states can be calculated by solving the quantum mechanical equation. In the actual experiment for the effect of nanobumps, the reduced dissociation energy of the water molecules can be larger than 0.323 eV because the radius of curvature of the nanobumped (or apical) surfaces might be larger than 0.5 nm (e.g., approximately, 5 nm in Fig. 2(d)). However, that value was considered to be considerably smaller than 5.03 eV for the flat surface because the apical radius of the curved surface was still extremely small (typically approximately 10 nm). Additionally, an illumination, e.g., a typical UV light (λ = 350 nm, 3.54 eV), further enhanced the dissociation of water molecules on the nanobumped surface because the photon energy was considerably larger than the dissociation energy for the nanobumped surface (0.323 eV). This energy difference resulted in the localised SPSC reaction increment via a photo-electron excitation effect, namely, the enhancement of equation (1) for the generation of additional excited electrons. Therefore, for the dissociated state of water molecules, which was induced by illumination, followed by an equilibration process, we obtained a final state, as shown in Fig. 3(c), in which H_2_ molecule (gaseous) formation was predicted.

### OH radical analysis

During the UV light irradiation of the plasma-treated Zn plate, 4 ml of PNDA with a concentration of 1.5 mg/l was used as a scavenger in OH radical detection. Based from estimated O_2_ production in Fig. 4(e), the greater factor of ~24 from the experiment results can neglect the dissolved gas effect in generated radicals: photo-induced water splitting governed the OH radical production. The absorption spectra before and after UV irradiation were compared: the concentration of PNDA was measured using a JASCO V-630 UV-Vis spectrophotometer. Then, the time vs. exponential decay was plotted, and the first-order reaction rate (k) was calculated.

### H_2_ gas analysis

The gas captured after UV_120h_ of six plasma-treated Zn substrates was used for GC analysis. The analysis of H_2_ and O_2_ gases was performed using a Shimadzu GC 8-A (thermal conductivity detector, molecular sieve 13X, N_2_ carrier for H_2_, and He carrier for O_2_). For H_2_ gas detection, 100% H_2_ gas (100 μl) was injected into the GC using a microsyringe, and the calibration curve was plotted. Then, 100 μl of the captured gas was injected into the GC, and its concentration was compared with the calibration curve. The same steps were repeated when recording the O_2_ concentration. The obtained result gives in the H_2_/O_2_ ratio of 10. The value is five times more than two in the case of normal water splitting.

## Additional Information

**How to cite this article**: Jeem, M. *et al.* A pathway of nanocrystallite fabrication by photo-assisted growth in pure water. *Sci. Rep.* **5**, 11429; doi: 10.1038/srep11429 (2015).

## Supplementary Material

Supplementary Information
