# Supplementary material for: A pathway of nanocrystallite fabrication by photo-assisted growth in pure water
Source: Sci Rep. 2015 Jun 16;5:11429. doi: 10.1038/srep11429 (PMC4468591; doi:10.1038/srep11429)
Supplement: Supplementary Information [file srep11429-s1.pdf]

A pathway of nanocrystallite fabrication by photo-assisted growth in pure water

Melbert Jeem[1], Muhammad Rafiq Mirza bin Julaihi[1], Junya Ishioka[2], Shigeo Yatsu[1,2], Kazumasa Okamoto[1,2], Tamaki Shibayama[1,2], Tomio Iwasaki[3], Takahiko Kato[2,3] & Seiichi Watanabe[1,2*]

1. *Graduate School of Engineering, Hokkaido University, N13, W8, Kita-ku, Sapporo, Hokkaido 060-8628, Japan*

2. *Faculty of Engineering, Hokkaido University, N13, W8, Kita-ku, Sapporo, Hokkaido 060-8628, Japan*

3. *Hitachi Research Laboratory, Hitachi Ltd., 7-1-1 Omika, Hitachi, Ibaraki, 319-1292, Japan*

Supplementary Figure S1.

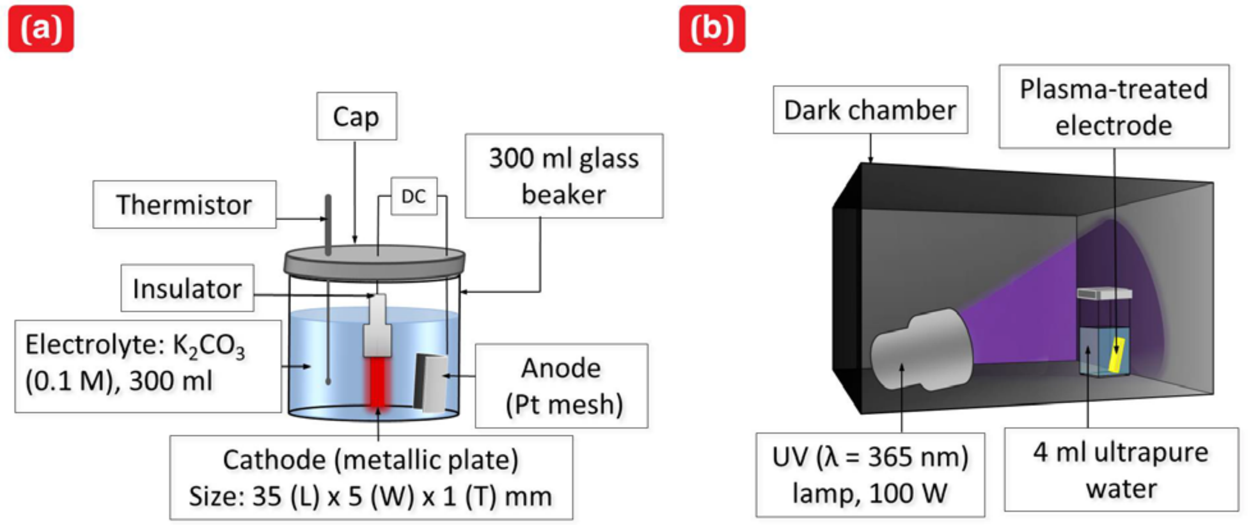

SPSC experimental setups. (**a**) Submerged liquid plasma experimental setup. (**b**) Submerged UV irradiation experimental setup.

Supplementary Figure. S2.

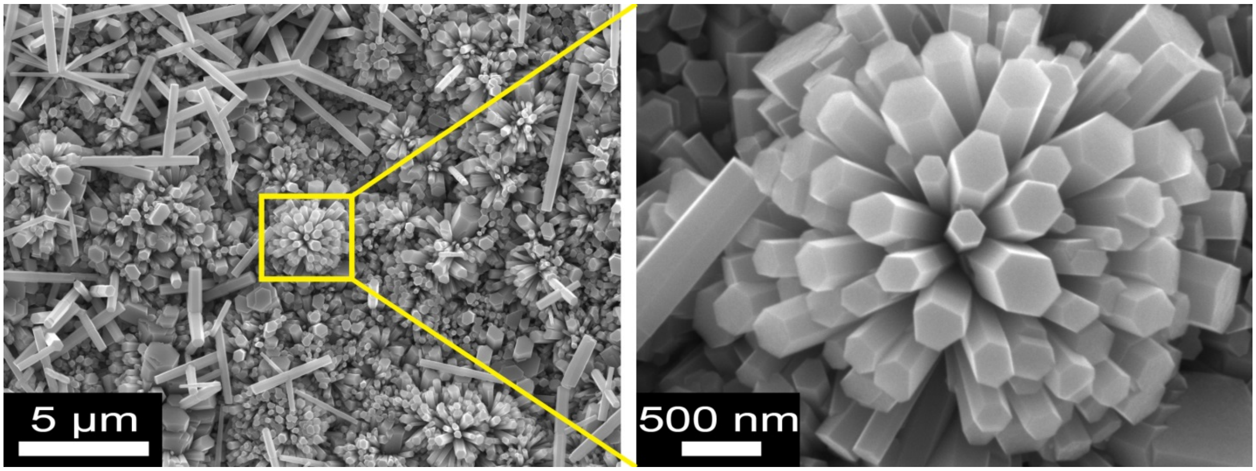

Terminated apical growth of ZnO nanoflowers. A continuous 72 h of UV irradiation in ultrapure water resulted in flat, hexagonal tips of the nanostructures. The right panel is the magnified FE-SEM image.

Supplementary Figure S3.

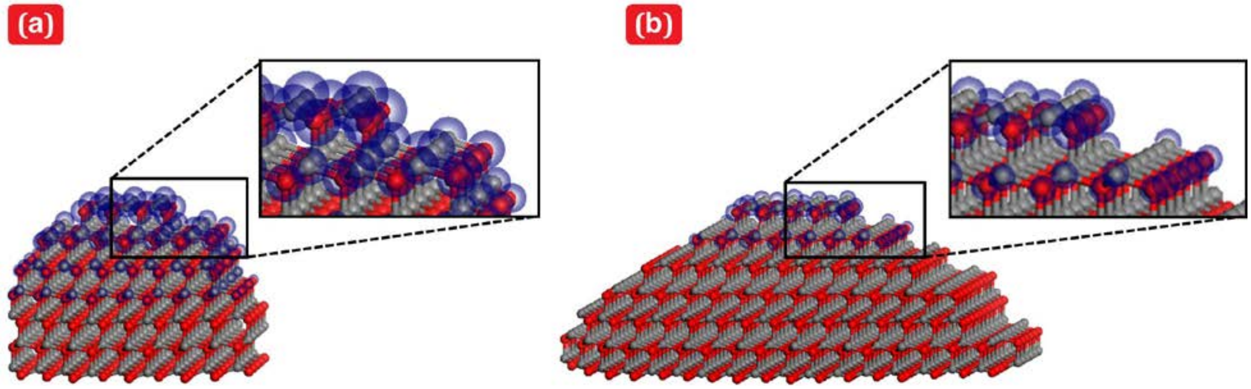

Curvature radius dependence of the 1.7 electron/Å^3 electron density isosurfaces for nanobumped ZnO. (**a-b**) R = 1.0 and 2.0 nm, respectively. The isosurfaces are indicated by the purple coloured region. The larger radius of curvature resulted in decreased electron density at the apex.
